# REALMS study: real-world effectiveness and safety of fingolimod in patients with relapsing-remitting multiple sclerosis in Portugal

**DOI:** 10.1007/s10072-020-04726-6

**Published:** 2020-09-30

**Authors:** S. Batista, C. C. Nunes, J. J. Cerqueira, Ana Martins Silva, J. Correia de Sá, J. Ferreira, M. T. Mendonça, J. Pinheiro, V. Salgado, A. S. Correia, J. Sequeira, A. Costa, L. Sousa

**Affiliations:** 1 Centro Hospitalar e Universitário de Coimbra, Praceta Prof. Mota Pinto, 3000-075 Coimbra, Portugal; 2 Hospital de Braga, Braga, Portugal; 3 School of Medicine, University of Minho, Braga, Portugal; 4 Centro Hospitalar Universitário do Porto, Porto, Portugal; 5 Unidade Multidisciplinar Investigação Biomédica, Instituto Ciências Biomédicas Abel Salazar, Universidade do Porto, Porto, Portugal; 6 Centro Hospitalar Lisboa Norte E.P.E., Hospital de Santa Maria, Lisbon, Portugal; 7 Centro Hospitalar São João, Porto, Portugal; 8 Centro Hospitalar Vila Nova Gaia, Vila Nova de Gaia, Portugal; 9 Hospital Prof. Doutor Fernando Fonseca E.P.E., Amadora, Portugal; 10 Centro Hospitalar de Lisboa Ocidental E.P.E., Hospital de Egas Moniz, Lisbon, Portugal; 11 Centro Hospitalar Universitário de Lisboa Central E.P.E., Hospital Santo António dos Capuchos, Lisbon, Portugal; 12 Novartis Farma, Lisbon, Portugal

**Keywords:** Relapsing-remitting multiple sclerosis, Real-world study, Fingolimod, ARR, EDSS, Safety

## Abstract

**Background:**

Fingolimod, an oral sphingosine 1-phosphate receptor modulator, is approved by EMA for relapsing-remitting multiple sclerosis (RRMS).

**Objectives:**

To assess the effectiveness and safety of fingolimod in patients with RRMS in real-world clinical practice in Portugal.

**Methods:**

Retrospective, multicentre, non-interventional study, reporting 3 years follow-up of data collected from October 2015 to July 2016. Sociodemographic data and previous treatments at baseline and data regarding disease evolution, including number of relapses, annualised relapse rates (ARR) and Expanded Disability Status Scale (EDSS), were collected.

**Results:**

Two-hundred and seventy-five participants were enrolled in the REALMS study. Results showed that the main reason to switch to fingolimod was failure of previous treatment (56.7%) and only 3.6% were naïve patients. In the total population, there was a significant decrease in ARR of 64.6% in the first year of treatment, 79.7% in the second year and 82.3% in the third year, compared with baseline. More than 67.0% of patients had no relapses during the 3 years after switching to fingolimod. EDSS remained stable throughout the study.

**Conclusions:**

Therapy with fingolimod showed a sustained effectiveness and safety over the 3 years, particularly on patients switched from first-line drugs (BRACE). No new safety issues were reported.

## Introduction

Fingolimod belongs to a class of drugs that targets the sphingolipid-regulated signaling system, acting as a functional antagonist of the sphingosine-1-phosphate type 1 (S1P1) receptor immunomodulatory [1], although some authors consider it to be an immunosuppressant [2]. It is a prodrug that is phosphorylated by sphingosine kinases to its active form, phosphofingolimod [1]. S1P1 is expressed abundantly on T and B lymphocytes, and fingolimod induces its downregulation by sequestering T cells in lymph nodes. This action prevents these cells from infiltrating inflammatory lesions in the central nervous system (CNS) [3], and fingolimod has been shown to decrease the pro-inflammatory marker IL-6 [4].

Fingolimod has shown to be effective in all four key measures of disease activity (relapse rate, disability progression, magnetic resonance imaging activity, and brain volume loss) compared with placebo or intramuscular interferon beta-1a in three pivotal clinical trials [5–7], although no differences were detected in the time to the confirmed progression of disability in the TRANSFORMS study, given its short duration (12 months) [6].

Fingolimod (Gilenya®) is the first approved oral disease-modifying therapy (DMT) worldwide. In the European Union [8], as well as in Portugal [9], fingolimod was approved as a single DMT in adult and paediatric patients aged 10 years and older [10] with highly active relapsing-remitting multiple sclerosis (RRMS) despite a full and adequate course of treatment with at least one DMT or patients with rapidly evolving severe RRMS defined by two or more disabling relapses in one year, and with one or more gadolinium-enhancing lesions on brain magnetic resonance imaging (MRI) or a significant increase in T2 lesion load as compared with a previous recent MRI.

Several studies of fingolimod in real-world populations have been published [11–15] confirming its effectiveness and safety shown in clinical trials in a more heterogeneous population.

The aim of the present study was to provide further data on the effectiveness and safety of fingolimod in a real-world clinical setting, for treatment-naïve and pre-treated patients with RRMS in Portugal.

## Methods

### Study design

REALMS was a retrospective, multicentre, national non-interventional study. Medical records were used to collect real-world evidence for the effectiveness and safety of fingolimod, as well as demographic and clinical characteristics of these patients. The study was conducted according to the tenets of the Declaration of Helsinki in its latest amendment (Brazil, 2013) and was approved by the Ethics Committee of each participating centre.

### Setting and participants

REALMS was a Portuguese multicentre study that retrospectively collected real-world evidence on the effectiveness, tolerability and safety from the clinical records of RRMS patients under fingolimod treatment in the most representative Portuguese MS centres. This paper reports 3-year follow-up of data collected from the 9 participating centres throughout Portugal from October 2015 to July 2016.

Inclusion criteria were as follows: age 18 years or more; diagnosis of RRMS according to the McDonald Criteria from 2010 [16]; patients who had initiated treatment with fingolimod at least 12 months before study enrolment, including those previously treated with interferon-β and/or glatiramer acetate, natalizumab or treatment naïve; at least 12 months of follow-up after initiating fingolimod treatment and sufficient data available on the clinical files. Treatment could have been discontinued, either temporarily or permanently, during these 12 months of follow-up (in case of temporary discontinuation, date of treatment initiation has been considered the date of the first administration of fingolimod); and patients that accepted to participate in the study and provided written informed consent to collect and analyse their data.

Exclusion criteria were patients who had previously been treated with fingolimod in a clinical trial before the inclusion in this study and had progressive course of MS (either secondary or primary progression) at the date of fingolimod treatment initiation.

### Collected variables and definitions

All variables were collected on an eCRF specifically designed for the study. The following data were obtained for all patients at baseline (12 months after initiation of fingolimod): age, sex, disease duration, date of first relapse, prevalence of other symptoms of MS, previous DMTs, duration of previous treatment with interferon-β or glatiramer acetate or natalizumab, reasons to switch to fingolimod, annualised relapse rates (ARR) and Expanded Disability Status Scale (EDSS) before switching to fingolimod, relevant comorbidities, and concomitant treatments. At follow-up, the following variables were collected: number of relapses, days to first relapse, ARR, EDSS, relevant comorbidities, treatment with costicosteroids, adverse events (AEs), fingolimod discontinuation, when applicable, and reason for discontinuation. A relapse was defined as patient-reported symptoms or objectively observed signs typical of an acute inflammatory demyelinating event in the CNS, current or historical, with duration of at least 24 hours, in the absence of fever or infection [16]. Progression of disability was defined as a 1-point increase in the EDSS score (or a half-point increase for patients with a baseline score above 5.0) that was confirmed at 6 months for up to 24 months [17].

### Quantitative variables and groups

In addition to descriptive statistics, inference statistics was performed in the whole population and three subgroups depending on treatment previous to fingolimod: naïve, interferon-β/glatiramer acetate or natalizumab. ARR and EDSS in the previous year and during the first 12, 24 and 36 months of treatment with fingolimod were compared. The proportion of relapse-free patients was also compared between these time points. The difference between these variables over time, within the same group, was also assessed.

### Safety assessments

The safety assessments analysed were as follows: descriptives of AEs during the first 24 h of the first fingolimod administration; description of AEs occurring after fingolimod treatment initiation; and maintenance of treatment during the first year of treatment with fingolimod. Disease activity, e.g. relapses or progression, were considered AEs.

### Statistical methods

An intent-to-treat (ITT) statistical analysis was performed. According to the original statistical analysis plan (SAP), statistical analysis was performed with available data, and no method of imputation for missing data was used. Quantitative variables were tested for normality using the Shapiro-Wilk test. Since the majority of quantitative variables were not normally distributed, baseline median, interquartile range (IQR), minimum and maximum are presented. For categorical variables, number and percentage of total are presented. Within-group analyses for quantitative variables were performed using the Friedman test and adjusted for multiple comparisons using the Sidak correction. For categorical variables, Cochran’s Q statistics or McNemar’s test were used as appropriate. The Kendall’s W test was used to analyse correlations. Between-group analyses for quantitative variables were performed using the Kruskal-Wallis test and adjusted for multiple comparisons using the Sidak correction. For categorical variables, the *χ*^2^ test was used. The Spearman *ρ* or Kendall’s *τ*-*b* were used to analyse correlations as appropriate. Predictors of EDSS at 3 years and ARR at 1, 2 and 3 years after switching to fingolimod were analysed using multivariate regressions using the backward conditional method. The dependent variable on the EDSS model was EDSS at 3 years, and the independent variables were sex, age, ARR in the year before switching to fingolimod, number of previous DMTs and years until switch to fingolimod. On all ARR models, the independent variables were the same as the ones included in the EDSS model plus: (1) baseline EDSS for the dependent variable ARR at 1 year; (2) baseline EDSS and ARR at 1 year for the dependent variable ARR at 2 years; and (3) baseline EDSS, ARR at 1 year and ARR at 2 years for the dependent variable ARR at 3 years. EDSS at baseline was not included in the predictor model of EDSS since these variables were significantly correlated (*n* = 37, Spearman *ρ* = 0.754, *p* = 0.01). A significance level of *α* = 0.05 was used (two sided). The software used was the SPSSv20.0 statistical package.

## Results

### Patient population

The REALMS study included 275 participants. All tables and figures state the number of patients with data available for each analysed variable and each time point. Missing data were considered to be missing completely at random (MCAR). Forty-one (14.9%) patients discontinued treatment with fingolimod during the study, twenty-seven (9.8%) of which permanently and fourteen (5.1%) temporarily. Therefore, the overall attrition rate of enrolled patients was 9.8%.

### Demographics and clinical baseline characteristics

Demographic and clinical characteristics of the total population and the three subgroups are described in Table 1. The 13 patients missing from Table 1 had previous treatments other than BRACE or natalizumab (off-label azathioprine (*n* = 4), mitoxantrone (*n* = 4), Ig IV (*n* = 4) and methotrexate (*n* = 1)).

**Table 1:** Demographics and clinical characteristics at fingolimod treatment initiation

Characteristic	Total cohort (*n* = 275)	Prior interferon-β or glatiramer acetate (*n* = 169)	Prior NTZ (*n* = 83)	Naïve (*n* = 10)
Female (*n* (%))	179 (65.1)	114 (67.5)	52 (62.7)	5 (50.0)
Disease duration (year; median (IQR))	10.0 (9.0; *n* = 273)	10.0 (8.0; *n* = 167)	12.0 (7.0)*	4.5 (9.0)**
Age (year; median (IQR))	41.0 (12.0)	43.0 (14.0)	40.0 (11.0)	36.5 (11.0)
EDSS score (median (IQR))	3.0 (2.0; *n* = 150)	2.5 (2.0; *n* = 88)	3.5 (2.5; *n* = 48)	2.8 (2.5; *n* = 8)
Prior treatments (median (IQR))	2.0 (2.0)	2.0 (2.0)	3.0 (1.0)	N/A

Data regarding subgroups by prior therapy analysed the last therapy before switching to fingolimod. The 13 patients missing had previous treatments other than BRACE or natalizumab (off-label azathioprine (*n* = 4), mitoxantrone (*n* = 4), Ig IV (*n* = 4) and methotrexate (*n* = 1))

*NTZ*, natalizumab; *N/A*, not applicable; *IQR*, interquartile range; *n*, number of patients

*p = 0.021 compared with interferon-β or glatiramer acetate; **p < 0.05 compared with interferon-β or glatiramer acetate and NTZ

The majority of patients were women (~ 65%). The median age at diagnosis for the total cohort and subgroups was between 40.0 and 43.0 years old, with the exception of the naïve subgroup who was younger (36.5 years). The median (IQR) disease duration was 10.0 (9.0) years. As expected, the naïve sub-group presented significant less years of disease duration compared with the interferon-β/glatiramer acetate and natalizumab sub-groups (4.5 vs. 10.0 and vs. 12.0 years, respectively, *p* < 0.05).

### Reasons to switch to fingolimod

The reasons to switch to fingolimod therapy were, in decreasing order of frequency, failure of previous treatment (56.7%), natalizumab withdrawal due to progressive multifocal leukoencephalopathy risk (35.3%) or other, such as adverse events from other treatments (6.2%). The remaining 3.6% were naïve patients with rapid disease progression, who initiated fingolimod as first-line therapy.

### Annualised relapse rate

**Fig. 1:**
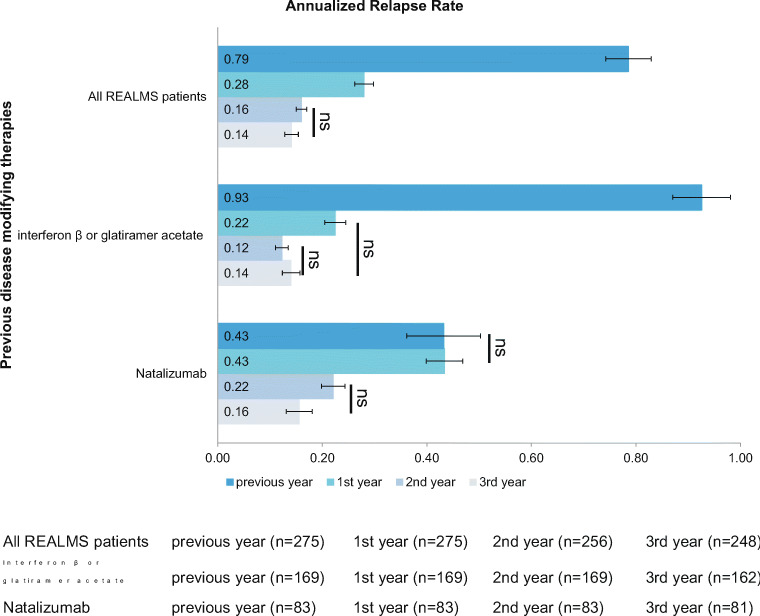
ARR before and after fingolimod therapy in the whole REALMS population and according to previous disease-modifying therapies. Error bars represent 95% CIs. See text for *p* values of comparisons not marked with ns

Considering the total population, there was a 64.6% decrease in ARR (0.79 vs. 0.28, *p* < 0.001) in the first year of treatment (Fig. 1). ARR further decreased by 79.7% in year 2 and 82.3% in year 3 compared with baseline (0.79 vs. 0.16 and vs. 0.14, respectively, *p* < 0.001). Patients previously treated with interferon-β or glatiramer acetate therapies showed an ARR reduction of 76.3% from baseline to year 1 post-fingolimod treatment (0.93 vs. 0.22, *p* < 0.001). The mean ARR decreased by 87.1% in year 2 (0.12, *p* < 0.001) and 84.9% in year 3 (0.14, *p* < 0.001) compared with baseline. Patients previously treated with natalizumab (*n* = 83) did not show a significant reduction of ARR in the first year of treatment. However, in these patients, ARR decreased by 48.8% in year 2 (0.43 vs. 0.22, *p* < 0.002) and 62.7% in year 3 (0.43 vs. 0.16, *p* < 0.001) compared with baseline.

Therapy with fingolimod showed a sustained effectiveness over the 3 years.

### Expanded Disability Status Scale

**Fig. 2:**
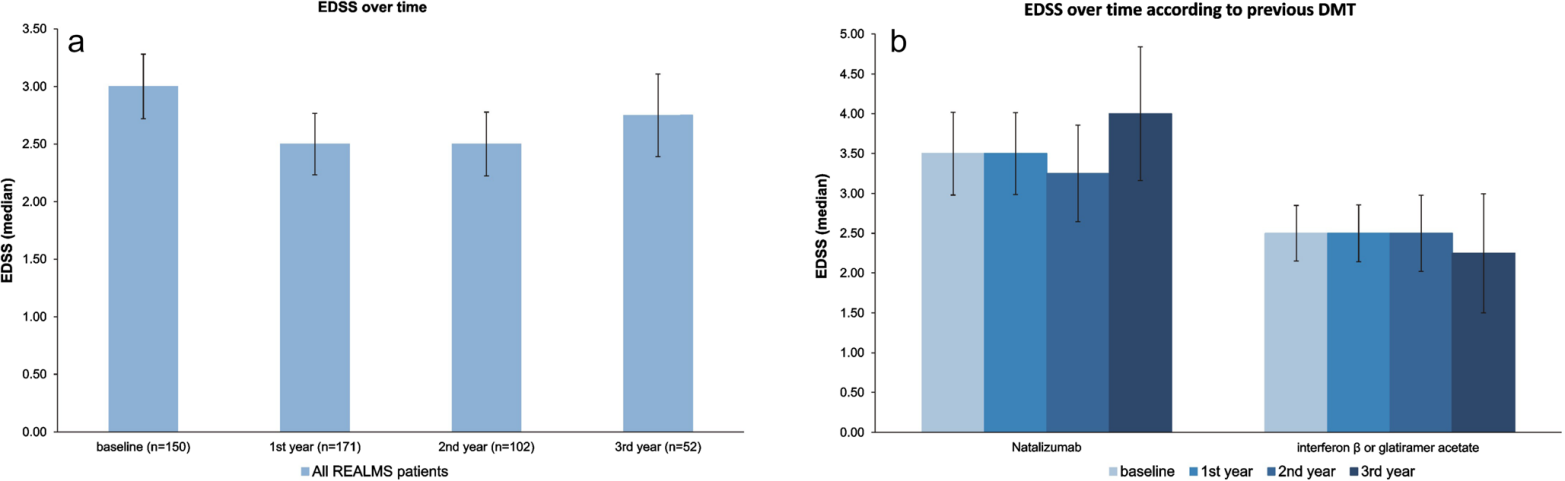
EDSS over time. Error bars represent 95% CIs. **a** Including all REALMS patients. *p* = ns for all comparisons. **b** According to previous disease-modifying therapies (DMTs). *p* = ns for all comparisons

The median baseline EDSS in the whole REALMS population was 3.00, changed to 2.50 after 1 year of fingolimod treatment (*p* > 0.05) and remained stable up to the end of the 3-year follow-up (Fig. 2a). When analysing by previous DMTs, both the previous interferon-β or glatiramer acetate and previous natalizumab groups remained with a stable EDSS over the 3 years of fingolimod therapy (Fig. 2b). Between group analyses showed that the interferon-β or glatiramer acetate group presented a significantly lower median EDSS compared with natalizumab 1 year after fingolimod therapy (*p* < 0.05), with both sub-groups remaining stable without differences between them after 2 and 3 years of fingolimod therapy.

### Relapse-free patients

Considering all patients of the REALMS study, 78.9 to 90.5% were relapse free in each of the 3 years of this study. From years 1 to 2, relapse-free patients significantly increased (78.9 vs. 87.3%, *p* = 0.016) and from years 1 to 3, further increased to 90.5% (*p* < 0.001). More than 67.0% of patients had no relapses during the 3 years after switching to fingolimod.

### Adverse events

There were a total of 61 (22.2%) AEs in 49 (17.8%) patients (Table 2). AEs were mostly related to disease activity (5.5%), followed by reductions in lymphocyte counts, opportunistic infections, increased liver enzymes and hypertension. None of these AEs were classified as serious AEs (SAEs).

**Table 2:** Adverse events by year after initiating fingolimod treatment

Adverse event	Year 1	Year 2	Year 3	Total by AE
	*N* (%)	*N* (%)	*N* (%)	*N* (%)
Opportunistic infections	2 (0.7)	3 (1.1)	1 (0.4)	9 (3.3)^a^
Grade 4 lymphopenia (< 200 cells/μl)	8 (2.9)	3 (1.1)	0 (0.0)	11 (4.0)
Hepatic enzymes > 3 × ULN	5 (1.8)	2 (0.7)	0 (0.0)	8 (2.9)^a^
Hepatic enzymes > 5 × ULN	1 (0.4)	0 (0.0)	0 (0.0)	1 (0.4)
Hypertension	0 (0.0)	1 (0.4)	1 (0.4)	2 (0.7)
Elevation of blood pressure	0 (0.0)	2 (0.7)	0 (0.0)	2 (0.7)
Disease activity (relapses, progression)	9 (3.3)	2 (0.7)	3 (1.1)	15 (5.5)^a^
Headaches	3 (1.1)	0 (0.0)	0 (0.0)	3 (1.1)
Other	8 (2.9)	2 (0.7)	0 (0.0)	10 (3.6)
Total by year	36 (13.1)	15 (5.5)	5 (1.9)	

*N*, number of patients; *%*, percentage of total; *ULN*, upper limit of normal

^a^Three opportunistic infections, one hepatic enzymes > 3 × ULN and one disease activity could not be assigned to a specific year given the dates are lacking

### Multivariate regression analyses

Predictors of EDSS at 3 years and ARR at 1, 2 and 3 years after switching to fingolimod were analysed with multivariate regressions using the backward conditional method (Table 3). Age in years and years until switch to fingolimod were positive predictors of EDSS at 3 years. Prior number of DMTs was a positive predictor of ARR at 1 year, and ARR at 1 year was a positive predictor of ARR at 2 years. None of the variables was a predictor of ARR at 3 years.

**Table 3:** Predictors of EDSS at 3 years and ARR at 1, 2, and 3 years after switching to fingolimod

Predictors	EDSS 3 years			ARR 1 year			ARR 2 years			ARR 3 years		
	Exp(B)	95% CI	*p* value	Exp(B)	95% CI	*p* value	Exp(B)	95% CI	*p* vale	Exp(B)	95% CI	*p* value
Age (years)	1.076	1.023–1.131	0.005									
Years until switch to fingolimod	1.155	1.038–1.287	0.010									
ARR 1 year							1.077	1.014–1.143	0.017	–	–	–
Prior DMTs (*n*)				1.028	1.008–1.050	0.007						

Statistically non-significant variables are not reported in the final model

*95% CI*, 95% confidence interval for Exp(B); *DMT*, disease modifying therapies

## Discussion

This study aimed to characterise the patients’ profile treated with fingolimod in the Portuguese real-word clinical practice, as well as to assess its effectiveness and safety.

In Portugal, the effectiveness and safety of fingolimod in a real-word population was previously studied in two single-centre studies [11, 18]. A multicentric study was necessary to contribute to the validation of the reported data. In these two studies, the reported discontinuation rate was 10.6% [11] and 15.6% (at 12 months) [18]. These attrition rates are similar to the ones found in the present study (9.8%), which are also similar to the ones reported by the real-word study with fingolimod from UK (approximately 8%) [13]. In other two real-world studies, one conducted in the Czech Republic, the GOLEMS study, 11.3% of patients discontinued fingolimod at or before 12 months [19] and another one conducted in Spain, the MS NEXT, 3.9% of patients permanently discontinued fingolimod during the first year of treatment [20].

Overall, the proportion of relapse-free patients significantly increased from 78.9 to 90.5% from the first to the third year after switching to fingolimod therapy. Moreover, 67% of the patients had no relapses during the 3 years after switching to fingolimod. These results are in accordance with the findings by Mazibrada et al and the MS NEXT study that reported 83.7% relapse-free patients 12 months after fingolimod initiation [13], and 67% relapse-free patients after two years of fingolimod treatment [20], respectively. The GENIUS study concluded that fingolimod appeared to be effective in naïve patients and after first-line treatment failure in reducing risk of relapse and disease activity throughout a 2-year follow-up [21]. In the two Portuguese real-world studies, the percentage of relapse-free patients at year 1 after switching to fingolimod was 75% [11] and 80.4% [18]. However, in this last study, the percentage of relapse-free patients decreased from years 1 to 3 of fingolimod treatment. Nevertheless, the percentage of patients with no relapses during the 3 years of fingolimod treatment was 60.8% [18], similar to that observed in our study.

Considering the total population and the group switching from interferon-β/glatiramer acetate, ARR significantly decreased in the first year of treatment with fingolimod and remained lower than baseline over 3 years. These results are in accordance with those reported by the PANGAEA study after 5 years of fingolimod therapy [22], a study conducted with similar methodological characteristics as the current study [23]. These results are also consistent with previously reported Portuguese studies whose results also showed a decrease in ARR in the first year of treatment with fingolimod [11, 18] and with the MS NEXT study that reported a 76% decrease in ARR after 2 years of fingolimod [20].

Mazibrada et al. reported that ARR in patients switching from natalizumab to fingolimod significantly decreased after the first year of fingolimod treatment [13], and the PANGAEA study at 4 years showed a decrease in ARR after the first year of fingolimod treatment that was maintained through 4 years, compared with baseline [24]. Our results showed that in the group of patients switching from natalizumab, there was no reduction of ARR in the first year of fingolimod treatment, although ARR decreased in years 2 and 3 compared with baseline. These results are in line with the ones reported by the two Portuguese real-world studies [11, 18].

Regarding disability, and as observed in the majority of studies [22, 25–27], the median value of EDSS at baseline was 3.0. Within-group analyses over time showed that EDSS remained stable over the 3 years after switching to fingolimod regardless of previous interferon-β/glatiramer acetate therapies. These results are in line with the FREEDOMS II and TRANSFORMS trials [6, 28]. Recently published results of real-world studies also showed a stable EDSS after switching to fingolimod, regardless of previous therapy [11, 13, 18, 22, 24].

Disability progression independent of relapse activity (PIRA) has been described as a frequent phenomenon in patients classified as RRMS. As a matter of fact, the term silent progression was recently proposed to describe the insidious disability that accrues in many patients who satisfy traditional criteria for RRMS. Therefore, we think that these results reflect precisely this phenomenon, since not all patients included in this cohort fulfil the criteria for secondary progressive MS. This suggests that the same process that underlies SPMS likely begins far earlier than it is generally recognised and supports a unitary view of MS biology, with both focal and diffuse tissue destructive components, and with inflammation and neurodegeneration occurring throughout the disease spectrum [29].

Interestingly, between-group analysis showed that the interferon-β/glatiramer acetate group presented a significantly lower median EDSS compared with natalizumab 1 year after fingolimod therapy and remained stable over the ensuing 2 years. These results have not been shown in the FREEDOMS, FREEDOMS II and TRANSFORMS trials [5, 6, 28], and are most probably a consequence from the fact that most patients switched to fingolimod based on clinical activity (relapses) in close temporal association with the time of switch. In line with this hypothesis, EDSS decreases were mostly due to switches from interferon-β/glatiramer acetate and not observed in the group that switched from natalizumab, in which the primary reason for the switch was not lack of efficacy, but being seropositive for the John Cunningham virus.

On multivariate regression analysis, the higher the age and the years until switch to fingolimod the higher the EDSS after 3 years. Also, the higher the number of previous DMTs the higher the ARR at year 1 after fingolimod initiation, and the higher the ARR at 1 year the higher the ARR at 2 years after fingolimod initiation. Taken together, and from a clinical standpoint, the faster the switch to fingolimod the better the results for the patient.

A sensitivity analysis for EDSS predictors at 3 years considering only patients that completed the 3-year follow-up (*n* = 52) has been performed. Age remained a positive predictor, although with slightly different values (Exp(B), 1.111; 95% CI, 1.043–1.183; *p* = 0.002), years until switch to fingolimod was no longer a predictor of EDSS at 3 years but prior DMTs were (Exp(B), 1.528; 95% CI, 1.021–2.289; *p* = 0.040). In fact, these two variables have the same interpretation and the conclusion to be drawn is the same: the early treatment with fingolimod is the real predictor of EDSS at 3 years, regardless if it is measured by proxy in years until switch to fingolimod or in prior DMTs. Therefore, this sensitivity analysis strengthens our results and conclusions.

In line with trials and real-world data, we observed no significant differences in ARR 1 year after switching from natalizumab to fingolimod whereas when changing from previous interferon-β/glatiramer acetate, the improvement of ARR at 1 year was evident.

In our population, and similar to other studies [11, 15, 18, 19, 30], fingolimod has shown to have a good safety profile. The proportion of patients discontinuing treatment was mostly related to disease activity (5.5%).

In RRMS, inflammation and the consequent presence of lymphocytes in the CNS is a hallmark of the disease, occurring in all its stages and courses. Therefore, new therapies that could act as promoters of the redistribution of lymphocytes back into circulation may reduce the immunomodulated axonal attack [31]. Thereby, previous treatment could hinder baseline parameters of inflammatory activity and future relapses induced by specific treatment. Perhaps this explains our results that showed that the time until switch to fingolimod was a predictor of higher EDSS at 3 years and a higher number of previous DMTs was a predictor of higher ARR at year 1 after fingolimod initiation.

## Limitations and strengths

REALMS had the inherent limitations of secondary data collection. There were too many missing MRI data to allow for an analysis of these parameters. Also, and although all investigators have been asked to record all adverse events, this is impossible to guarantee. One of the biggest strengths of this study was the large sample size. This was the largest observational study done up to date, involving 9 of the most important MS centres in Portugal.

## Conclusions

The REALMS study provides real-world data confirming the effectiveness and safety of fingolimod under real-world conditions, in the Portuguese population, consistent with phase 3 trials. Fingolimod is an effective treatment in real-world populations with RRMS regardless of previous DMTs. Moreover, significant reductions in relapses were seen after switching from interferon-β or glatiramer acetate to fingolimod, suggesting that these patients benefit from switching to fingolimod. Fingolimod revealed to be a good option treatment for the majority of patients after switch from natalizumab, since most of these patients were relapse free (88.9%) and free from progression of disability (66.7%) after 3 years of treatment with fingolimod. Furthermore, these data suggest that the earlier the treatment with fingolimod, the better the outcomes for patients with RRMS.
